# The use of the surgical pleth index to guide anaesthesia in gastroenterological surgery: a randomised controlled study

**DOI:** 10.1007/s10877-025-01262-6

**Published:** 2025-01-20

**Authors:** Tommi Bergman, Maija-Liisa Kalliomäki, Mika Särkelä, Jarkko Harju

**Affiliations:** 1 Tampere University Hospital, Tampere, Finland; 2 GE Healthcare Finland Oy, Helsinki, Finland

**Keywords:** General anaesthesia, Nociception, Surgical pleth index, Intraoperative hypotension

## Abstract

The measurement of nociception and the optimisation of intraoperative antinociceptive medication could potentially improve the conduct of anaesthesia, especially in the older population. The Surgical Pleth Index (SPI) is one of the monitoring methods presently used for the detection of nociceptive stimulus. Eighty patients aged 50 years and older who were scheduled to undergo major abdominal surgery were randomised and divided into a study group and a control group. In the study group, the SPI was used to guide the administration of remifentanil during surgery. In the control group, the SPI value was concealed, and remifentanil administration was based on the clinical evaluation of the attending anaesthesiologist. The primary endpoint of this study was intraoperative remifentanil consumption. In addition, we compared the durations of intraoperative hypotension and hypertension. No difference in intraoperative remifentanil consumption (4.5 µg kg^− 1^h^− 1^ vs. 5.6 µg kg^− 1^h^− 1^, *p* = 0.14) was found. Furthermore, there was no difference in the proportion of hypotensive time (mean arterial pressure, MAP < 65) (3.7% vs. 1.6%, *p* = 0.40). However, in the subgroup of patients who underwent operation with invasive blood pressure monitoring, there was less severe hypotension (MAP < 55) (0.3% vs. 0.0%, *p* = 0.02) and intermediate hypotension (MAP < 65) (10.2% vs. 2.6%, *p* = 0.07) in the treatment group, even though remifentanil consumption was higher (3.5 µg kg^− 1^h^− 1^ vs. 5.1 µg kg^− 1^h^− 1^*p* = 0.03). The use of SPI guidance for the administration of remifentanil during surgery did not help to reduce the remifentanil consumption. However, the results from invasively monitored study group suggest more timely administered opioid when SPI was used.

## Introduction

General anaesthesia consists of three components: hypnosis, immobility and antinociception [1]. All three components can be affected individually or in combination by administering anaesthetic drugs. Similarly, routine intraoperative monitoring comprises electrocardiogram (ECG), blood oxygen saturation, blood pressure, depth of relaxation and depth of anaesthesia. Thus, the measurement of nociceptive stimulus is not yet part of routine intraoperative monitoring and the benefits of nociception monitor-guided opioid administration remain unclear [2].

The number of older adults undergoing surgery has increased and this increase will continue [3]. In many cases, frail, older patients are more vulnerable to anaesthesia related and postoperative complications [4, 5]. Interestingly, for those older patients who undergo major surgery, optimisation of the depth of anaesthesia can reduce the risk of postoperative delirium and cognitive impairment [6]. Therefore, Surgical Pleth Index (SPI)-guided administration might serve to improve the quality of the anaesthesia [7]. In contrast, low remifentanil doses are associated with an increase in serum cortisol concentrations [8], and high cortisol levels may be associated with delayed wound healing and increased mortality [9].Therefore, older patients may benefit from the optimisation of opioid administration.

The SPI (GE HealthCare, Helsinki, Finland) is an intraoperative monitoring system for the detection of nociceptive stimulus that is based on the analysis of the photoplethysmographic waveform [10]. The system normalises the photoplethysmographic amplitude and heartbeat interval into an index number ranging from 0 to 100. The SPI value is calculated using the following equation: SPI = 100 − (0.7 × PPGAnorm + 0.3 × HBInorm), where PPGAnorm and HBInorm represent the normalised photoplethysmographic amplitude (PPGA) and the heartbeat interval (HBI) [11]. The SPI is high when nociceptive stimulation is high and analgesia is inadequate, whereas the SPI is low when analgesia is adequate [10]. The SPI does not require any disposable equipment. Most trials that have studied the SPI have used a target range of between 20 and 50^2^. In other words, when SPI value is high plethysmosgraphic amplitude has decreased, heart rate increased or both changed indicating nociceptive reaction and potential need for opioids. This may be compromised more with inhalational anaesthetics (such as sevoflurane) than with propofol, since they cause more vasodilatation than propofol [12]. Similar effect might also be seen, if vasoactive drugs are used extensively decreasing plethysmographic amplitude. SPI has still been shown to be effective apart from use of vasoactive agents [13] Furthermore, during anaesthesia, vasoactive agents are used to increase the blood pressure which is typically low if anesthesia is too deep. When the anesthesia is more shallow, blood pressures are higher and vasoactive agents are not used and there are less confounding factors for measurements such as SPI.

The aim of this randomised, controlled, single-blinded trial was to determine whether a target range of below 50 for SPI-guided remifentanil administration stabilises the conduct of anaesthesia and reduces the need for vasoactive drug administration and/or reduces unwanted anaesthesia related events in patients aged 50 years and older who are undergoing gastroenterological surgery.

## Materials and methods

The study was conducted at Tampere University Hospital, Finland between September 9, 2020 and November 5, 2021. The study was registered at (clinicaltrials.gov) (NCT04519203) prior to the commencement of the study. In addition, approval was obtained from the local ethics committee (Regional Ethics Committee of the expert responsibility area of Tampere University Hospital) (ETL R2006). All monitoring devices were CE marked and used in accordance with the labelled indications. Written informed consent was obtained from all patients prior to inclusion in the study.

### Inclusion criteria

A total of 80 patients were randomised into two equal groups. The included patients were aged 50 years and older, had an American Society of Anesthesiologists (ASA) classification of between 1 and 3 and were due to undergo laparoscopic or laparotomic surgery requiring intubation with an expected intraoperative time of at least two hours. The choice of age limit was made to rule out the younger age groups in the population. The exclusion criteria consisted of implanted cardiac pacemaker or known condition with irregular heart rate at the time of inclusion or induction, the chronic use of opioids, BMI > 35, known allergy for the study medications, more than 5 extrasystoles per minute at the time of induction or inclusion, the use of epidural catheter during the previous one hour prior to surgery or the need for a catheter intraoperatively.

### Conduct of the study

Before the start of anaesthesia, the patients were randomised into two groups, the SPI-guided group and the control group, using a sealed, blinded, and numbered envelope which contained the randomisation information. There were 40 patients in each group. In the SPI-guided group, the administration of opioids was guided using a SPI based protocol. In the control group, however, the administration of opioids was based on standard monitoring and the clinical evaluation of the attending anaesthesiologist alone. Furthermore, the SPI value displayed on the monitor was covered with an opaque piece of cardboard. The patients were premedicated using oral midazolam and 1 g paracetamol if necessary.

Patients were monitored using standard operation monitoring, which included ECG, saturation, relaxation, and entropy measurement. Blood pressure was measured using non-invasive blood pressure (typically every five minutes) or invasive arterial cannula. The decision on the type of measurement was made by the attending anaesthesiologist based on clinical decision mostly if the patient was considered unstable or if major blood loss was anticipated.

The anaesthesia was provided using target-controlled infusions of remifentanil (pharmacokinetic model: Minto, effect site [14]^)^ and propofol (model: Schnider, effect site [15]). The remifentanil limits were 1–8 ng ml^− 1^, and the propofol limits were 2–8 µg ml^− 1^. An exception to this was that the higher limit of remifentanil was ≤4ng ml^− 1^ when propofol was lowered to < 3 µg ml^− 1^.

In both study groups, the propofol infusion rate was guided by entropy measurement as targeted to state entropy (SE) values of between 40 and 60. The propofol was titrated by steps of 0.5 to 1.0 µg ml^− 1^ based on the clinical judgement of the attending anaesthesiologist. If the SE value was below the target range, the propofol was lowered using fluent changes at least 10 min after noticing the SE value was below the target range. Any new changes were made at least 5 min after each change if the target was not reached. Towards the end of the operation, the SE value could be increased to 65.

Prior to extubation the patients were treated with 1 g paracetamol i.v. if not contraindicated or given as premedication. In addition, 0,1 mg/kg oxycodone was given intramuscularly, and 2 mg intravenously. If an epidural was to be used, it was activated in the operation room after the final suture. The pain relief was given according to normal postoperative guidelines during the PACU mainly using oxycodone intravenously or per orally.

### Induction

At the induction, both infusions were started at 4 ng ml^− 1^ and 4 µg ml^− 1^. The SE was aimed to the target range shortly after intubation, but the SPI (in the SPI-guided group) was only considered after the start of surgery.

Muscle relaxation was maintained using rocuronium based on surgical needs (induction 0.6 mg kg^− 1^ and thereafter 10–30 mg once needed). Towards the end of the operation, the relaxation was allowed to fade, and any possible residual relaxation was reversed using Sugammadex 0.5-2 mg kg^− 1^ to reach a train-of-four (TOF) of > 90% prior to extubation.

Intraoperative fluid was administered using pumps infusing primarily Ringer-based solution 1–2 ml/kg/h. The possible losses were replaced using clear fluids, blood or anticoagulation products when necessary. The target value for haemoglobin was > 80 g l^− 1^.

Severe bradycardia (heart rate < 35/min) was treated with atropine in both groups (0.3–0.5 mg).

### The control group

In the control group, the administration of remifentanil was based on clinical decision. If the systolic blood pressure was over 140 mmHg, heart rate over 90/min or there was a 10 to 20% increase within a short period, the remifentanil concentration was changed. If SE was > 60, the propofol concentration was also raised simultaneously.

If either the mean blood pressure (MAP < 60–70 mmHg based on clinical decision) or heart rate were too low, primarily the remifentanil concentration was decreased. Regarding sedation status, propofol concentration was lowered if SE was < 40. Secondarily, blood pressure was raised using noradrenaline infusion or bolus. In cases where the blood pressure remained low or the need for noradrenaline increased, an additional fluid chosen by the attending anaesthesiologist was administered.

The central core temperature was followed and targeted at > 36 °C prior to extubation.

### The SPI-group

In the SPI-group, the administration of remifentanil was guided using the SPI (target < 50). Since the typical lower limit of 20 is mainly an indicator of deep anaesthesia, we decided not to include it in the protocol. Therefore, the indication of too deep anaesthesia was decided in another way, which is defined below. The dosage of remifentanil was changed in steps of 0.5-1.0 ng ml^− 1^ based on clinical decision. A high SPI number was reacted to latest within 10 min, and any additional changes were made within 5 min if the target was not reached. The remifentanil dosage was not decreased if the patient was hypertensive (systolic blood pressure over 140 mmHg) and not increased if the patient was hypotensive (mean blood pressure under 60–70 mmHg, based on clinical decision). If the patient’s clinical status mandated, the SPI was overruled, and the patient was treated based on clinical decision. However, this had to be marked as a protocol deviation and the exact reason for the decision had to be explained in the commentary.

If the patient was hypotensive (mean blood pressure < 60–70 mmHg based on clinical decision), primarily the remifentanil was decreased. Secondarily, blood pressure was raised using noradrenaline infusion or bolus. In cases where blood pressure remained low or the need for noradrenaline increased, a fluid chosen by the attending anaesthesiologist was administered.

### Study power and endpoints

The study power was calculated using a reference study that compared the depth of anaesthesia and the depth of nociception in a balance-controlled group to a group without these monitors. In the monitored group, the consumption of remifentanil was 9.5 ± 3.8 µg kg^− 1^ h^− 1^, whereas in the unmonitored group the consumption was 12.3 ± 5.2 µg kg^− 1^ h^− 1^ [16]. While considering that α = 0.05 and β = 90%, 39 patients were needed in each group. Therefore, we chose a total of 80 patients to be included in the study.

The primary endpoint of this study was intraoperative remifentanil consumption. Secondary endpoints were signs of inadequate anaesthesia such as the need for vasoactive medication, severe hypotension (MAP < 55) and intermediate hypotension (MAP < 65), bradycardia (HR < 45), tachycardia (HR > 90) and intraoperative propofol consumption (Table 1). Severe hypotension, intermediate hypotension, hypertension, tachycardia, and bradycardia were analysed by deriving the time spent below or above the predefined threshold value for each patient and dividing the result by surgery time (from first incision to wound closure). The instability of the anaesthesia was also measured using the hemodynamic instability score [17], and the severity of the illnesses was described using the Charlson Comorbidity Score [18].

The PACU treatment was given according to routine patient and operation-based protocols. The PACU treatment lasted at least two hours during which a modified Aldrete score and numeric rating scale (NRS 0–10) is followed at arrival and thereafter every 30 min by the PACU nurse. The data was collected until the patient was fit for discharge but maximum four hours after coming to PACU. The study was ended once the patient was fit for charge or the four hours timeline was reached.

### Statistics

The normality of the data was assessed with the Lilliefors test. For normally distributed data, mean and standard deviation are presented, and the differences between groups were assessed with *T* test. For non-normally distributed data, medians and inter-quartile ranges are presented, and the differences between groups were assessed with Mann-Whitney *U* test. Fisher’s exact test was used for data on three categorical variables (sex, type of surgery, PONV). For variables with more than two categories (activity level, ventilation, circulation, consciousness, oxygen saturation), the χ^2^ test [19] was used. A limit of statistical significance was set at α = 0.05. All statistics were conducted with MATLAB R2018b (The Mathworks Inc, Natick, MA, USA).

**Table 1:** Study endpoints

● Primary endpoints:
o Intraoperative remifentanil consumption
● Secondary endpoints:
o Signs of inadequate anaesthesia:
▪ The need for vasoactive medication
▪ Hypotension
● severe hypotension
o MAP < 55 mmHg,
● intermediate hypotension)
o MAP < 65 mmHg
▪ hypertension
● (Systolic pressure > 140 mmHg)
▪ Bradycardia (Heart rate < 45)
▪ tachycardia (Heart rate > 90)
o Intraoperative propofol consumption
o Time from end of surgery to extubation
o Numeric rating score during postoperative care treatment
o Disorientation/ grade of sedation during postoperative care treatment
o Postoperative nausea and vomiting

## Results

**Fig. 1:**
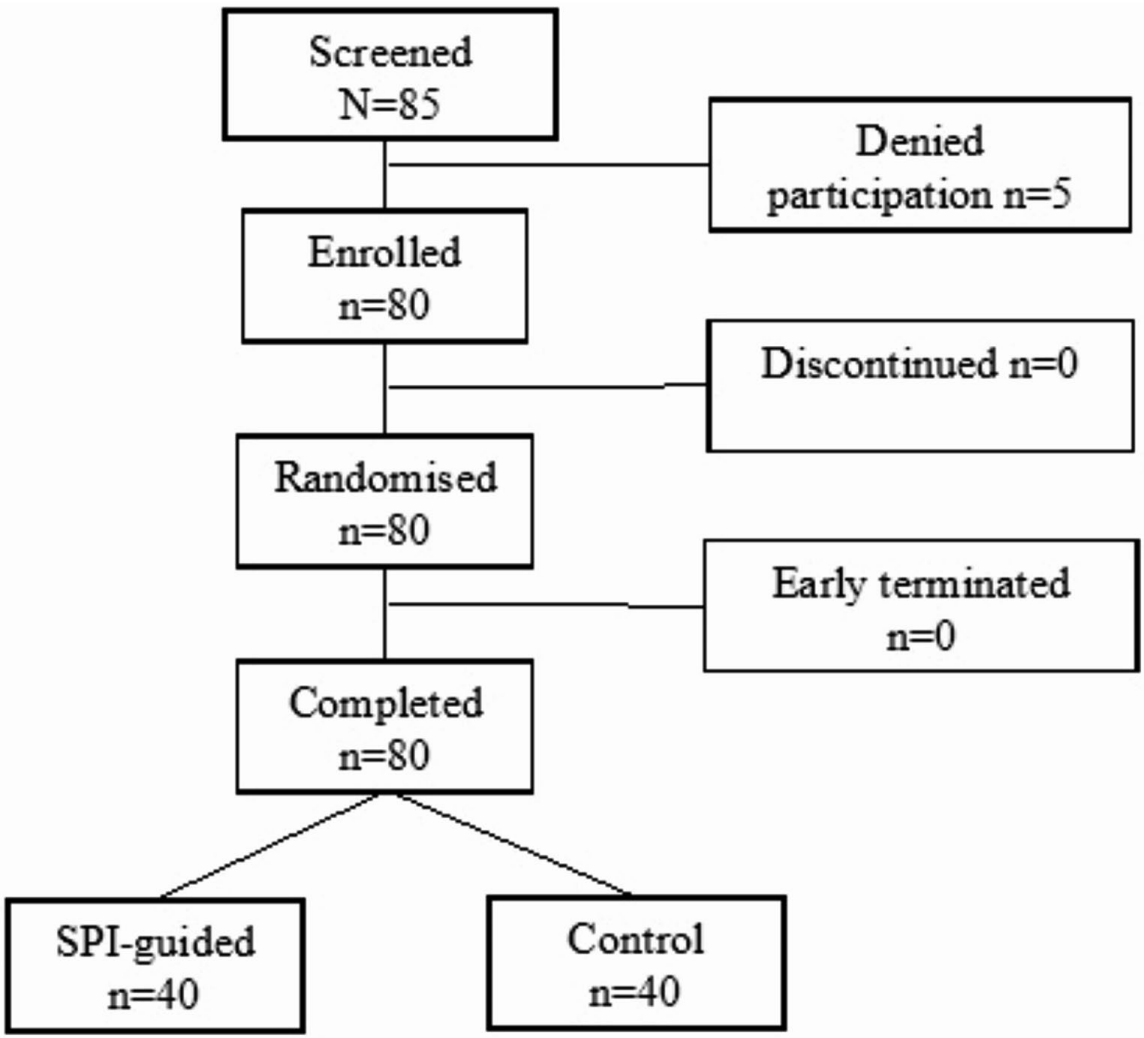
Flowchart of patient enrolment

A total of 80 patients were recorded successfully Fig. 1. There were no differences in the characteristics between groups. The mean age was 69.2 ± 8.9 years, and the patients had several comorbidities (median Charlson Comorbidity index 5). The type of surgery was mostly laparoscopy (Table 2).

**Table 2:** Characteristics of patients described as mean ± SD or number except for CCI and surgery duration as median [inter-quartile range], BMI = body mass index, CCI = Charlson comorbidity index

	All	Control	SPI	*p*
Sex				0.82
Female [n]	38	18	20	
Male [n]	42	22	20	
Age [yrs.]	69.2 ± 8.9	70.4 ± 8.6	68.0 ± 9.3	0.22
Height [cm]	171.3 ± 9.3	172.3 ± 10.7	170.3 ± 7.6	0.35
Weight [kg]	76.5 ± 14.1	77.6 ± 15.0	75.5 ± 13.3	0.51
BMI [kg/m2]	25.9 ± 3.6	26.0 ± 3.9	25.9 ± 3.3	0.83
ASA (I, II, III)	3/44/33	2/18/20	1/26/13	0.19
CCI	5 [3–6]	5 [4–7]	4 [3–5]	0.05
Type of surgery				1.00
Laparoscopy [n]	71	35	36	
Laparotomy [n]	9	5	4	
Surgery duration [HH: MM]	03:01 [01:58 − 03:44]	03:00 [01:58 − 03:54]	03:01 [02:00–03:40]	0.69

There was no difference in the intraoperative consumption of remifentanil between the two groups (Control 4.5 µg kg^− 1^ h^− 1^ [3.5–6.8] vs. SPI 5.6 µg kg^− 1^ h^− 1^ [4.2–6.9], *p* = 0.14). The overall percentage of time for MAP < 65 mmHg was relatively low (2.3%) and no difference was found between the groups (3.7 vs. 1.6%, *p* = 0.40). The characteristics of post-operative treatment did not differ between the groups (Table 3). The characteristics during post operative treatment did not differ between the groups (Table 4).

**Table 3:** Description of primary endpoints. Described as median [inter-quartile range] for anaesthetics, time fraction [inter-quartile range] for intraoperative values. Heart rate = HR, MAP = mean arterial psressure

	All	Control	SPI	*p*
Remifentanil [µg kg^− 1^ h^− 1^]	5.1 [3.8–6.9]	4.5 [3.5–6.8]	5.6 [4.2–6.9]	0.14
Propofol [mg kg^− 1^ h^− 1^]	6.3 [5.4–7.3]	6.2 [5.2–7.2]	6.5 [5.6–7.5]	0.34
Noradrenaline [µg kg^− 1^ h^− 1^]	0.44 [0.12–0.86]	0.47 [0.25–0.82]	0.36 [0.00-0.93]	0.36
Hypotension MAP < 55 [%]	0.0 [0.0-0.1]	0.0 [0.0-0.2]	0.0 [0.0–0.0]	0.37
Hypotension MAP < 65 [%]	2.3 [0.0-9.7]	3.7 [0.2–10.6]	1.6 [0.0-7.6]	0.40
Hypertension Systolic pressure > 140 [%]	3.3 [0.0-14.1]	2.3 [0.0-14.1]	3.5 [0.0-13.5]	0.62
Bradycardia HR < 45 [%]	0.0 [0.0-0.1]	0.0 [0.0-0.2]	0.0 [0.0–0.0]	0.31
Tachycardia HR > 90 [%]	0.0 [0.0-0.1]	0.0 [0.0-0.1]	0.0 [0.0-0.1]	0.83
Time to extubation [HH: MM]	00:11 [00:07 − 00:16]	00:10 [00:07 − 00:15]	00:13 [00:08 − 00:16]	0.19
Hemodynamic instability-score	15 [8–28]	17 [10–31]	14 [7–27]	0.19

**Table 4:** Characteristics of post anaesthesia care treatment at entry to PACU (A) and at the timepoint of one hour (B). (PONV = post-operative nausea and vomiting, NRS = numeric rating scale)

A.		All patients	Control	SPI	*p*
Activity Level					0.27
	Does Not Move Extremities	53.8% (42/78)	47.4% (18/38)	60.0% (24/40)	
	Moves Two Extremities	15.4% (12/78)	13.2% (5/38)	17.5% (7/40)	
	Able To Move Four Extremities	30.8% (24/78)	39.5% (15/38)	22.5% (9/40)	
Ventilation					0.37
	Apnoeic	2.5% (2/79)	0.0% (0/39)	5.0% (2/40)	
	Dyspnoea With Shallow Breathing	20.3% (16/79)	20.5% (8/39)	20.0% (8/40)	
	Spontaneous	77.2% (61/79)	79.5% (31/39)	75.0% (30/40)	
Circulation					0.25
	BP > ± 50% Pre-anaesthetic Level	7.5% (6/80)	7.5% (3/40)	7.5% (3/40)	
	BP ± 20 to 50% Pre-anaesthetic Level	36.3% (29/80)	27.5% (11/40)	45.0% (18/40)	
	BP < ± 20% Pre-anaesthetic Level	56.3% (45/80)	65.0% (26/40)	47.5% (19/40)	
Consciousness					0.55
	Not Responding	40.5% (32/79)	35.9% (14/39)	45.0% (18/40)	
	Aroused Upon Calling	43.0% (34/79)	43.6% (17/39)	42.5% (17/40)	
	Fully Awake	16.5% (13/79)	20.5% (8/39)	12.5% (5/40)	
Oxygen Saturation					0.62
	Requires O2 Inhalation to Maintain Level > 90%	95.0% (76/80)	92.5% (37/40)	97.5% (39/40)	
	Maintains Level > 90% in Room Air	5.0% (4/80)	7.5% (3/40)	2.5% (1/40)	
PONV yes		0.0% (0/67)	0.0% (0/37)	0.0% (0/30)	1.00
NRS median IQR		0 [0–3]	0 [0–3]	0 [0–2]	0.53
Activity Level					1.00
Does Not Move Extremities		0.0% (0/80)	0.0% (0/80)	0.0% (0/80)	
	Moves Two Extremities	1.3% (1/80)	0.0% (0/40)	2.5% (1/40)	
	Able To Move Four Extremities	98.8% (79/80)	100.0% (40/40)	97.5% (39/40)	
Ventilation					1.00
	Apnoeic	0.0% (0/80)	0.0% (0/80)	0.0% (0/80)	
	Dyspnoea With Shallow Breathing	6.3% (5/80)	5.0% (2/40)	7.5% (3/40)	
	Spontaneous	93.8% (75/80)	95.0% (38/40)	92.5% (37/40)	
Circulation					0.46
	BP > ± 50% Pre-anaesthetic Level	5.0% (4/80)	5.0% (2/40)	5.0% (2/40)	
	*P* ± 20 to 50% Pre-anaesthetic Level	28.8% (23/80)	35.0% (14/40)	22.5% (9/40)	
	BP < ± 20% Pre-anaesthetic Level	66.3% (53/80)	60.0% (24/40)	72.5% (29/40)	
Consciousness					0.35
	Not Responding	0.0% (0/80)	0.0% (0/80)	0.0% (0/80%)	
	Aroused Upon Calling	15.0% (12/80)	20.0% (8/40)	10.0% (4/40)	
	Fully Awake	85.0% (68/80)	80.0% (32/40)	90.0% (36/40)	
Oxygen Saturation					0.60
	Requires O2 Inhalation to Maintain Level > 90%	76.3% (61/80)	72.5% (29/40)	80.0% (32/40)	
	Maintains Level > 90% in Room Air	23.8% (19/80)	27.5% (11/40)	20.0% (8/40)	
PONV yes NRS median IQR		1.3% (1/80)	2.5% (1/40)	0.0% (0/40)	1.00
		2 [0–4]	2 [0–5]	2 [0–4]	0.64

**Fig. 2:**
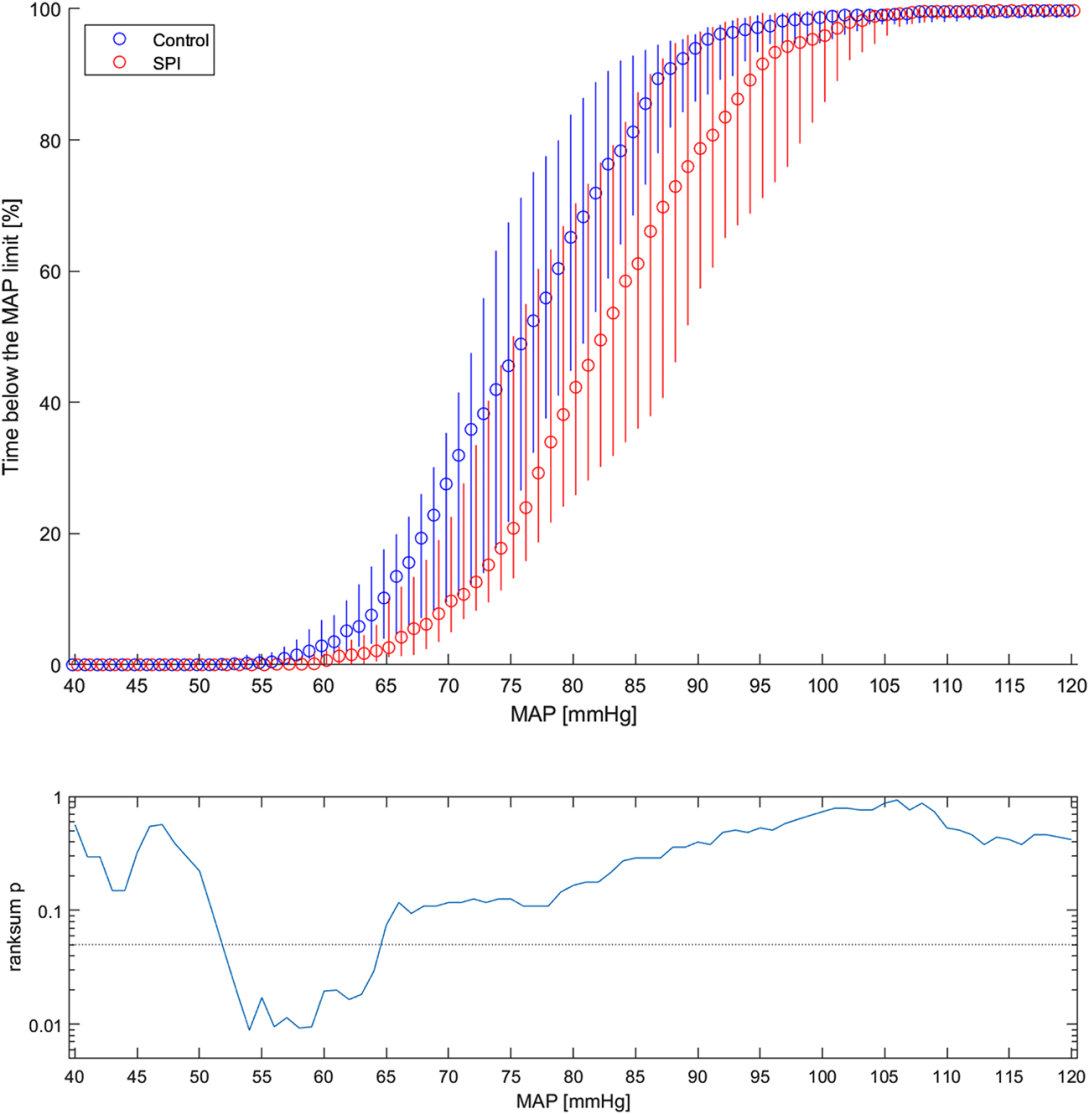
The graph at the top shows the medians (circles) and inter-quartile ranges (vertical lines) of fractions of the time spent below defined mean pressures in the subgroup of patients with invasive blood pressure measurement shown separately for the control group and the SPI group. The graph below presents the corresponding p-values of the Mann-Whitney U test

The patients were further divided into two subgroups based on the method of blood pressure measurement. In the invasively monitored group, the consumption of remifentanil was higher (3.5 µg/kg/h vs. 5.1 µg/kg/h, *p* = 0.03) in the treatment group, but the duration of severe hypotension (MAP < 55 mmHg) (0.3% vs. 0.0%, *p* = 0.02) and intermediate hypotension (MAP < 65) (10.2% vs. 2.6%, *p* = 0.07) was shorter than in the control group (Table 5) Fig. 2.

**Table 5:** Description of subgroup analysis. Described as median [inter-quartile range] for anaesthetics, and time fraction [inter-quartile range] for intraoperative values

		All	Control	SPI	*p*
Non-invasive (n)		47	23	24	
	Remifentanil [µg kg^− 1^ h^− 1^]	5.9 [4.2–7.2]	6.1 [4.1–7.3]	5.9 [4.2–6.9]	0.99
	Propofol [mg kg^− 1^ h^− 1^]	6.5 [5.5–7.5]	6.2 [5.5–7.3]	6.6 [5.6–7.7]	0.68
	Noradrenaline [µg kg^− 1^ h^− 1^]	0.26 [0.00-0.70]	0.35 [0.15–0.56]	0.21 [0.00-0.86]	0.52
	Hypotension MAP < 55[%]	0.0 [0.0–0.0]	0.0 [0.0–0.0]	0.0 [0.0–0.0]	0.17
	Hypotension MAP < 65[%]	1.1 [0.0-5.3]	1.0 [0.0-4.9]	1.2 [0.0-6.1]	0.80
	Hypertension SysBP > 140[%]	3.3 [0.0-17.8]	2.4 [0.0-21.9]	3.3 [0.0–9.0]	0.91
	Bradycardia HR < 45[%]	0.0 [0.0-0.5]	0.0 [0.0-0.2]	0.0 [0.0-0.8]	0.97
	Tachycardia HR > 90[%]	0.0 [0.0-0.1]	0.0 [0.0–0.0]	0.0 [0.0-0.1]	0.55
	Time To Extubation [HH: MM]	00:11 [00:07 − 00:15]	00:11 [00:06 − 00:14]	00:11 [00:07 − 00:15]	0.63
	Hemodynamic instability score	9 [6–13]	11 [7–13]	8 [5–13]	0.24
Invasive (n)		33	17	16	
	Remifentanil [µg kg^− 1^ h^− 1^]	4.3 [3.3–6.2]	3.5 [2.5–4.8]	5.1 [4.2-7.0]	0.03
	Propofol [mg kg^− 1^ h^− 1^]	6.0 [5.2–7.2]	6.0 [4.9–6.9]	6.1 [5.7–7.3]	0.27
	Noradrenaline [µg kg^− 1^ h^− 1^]	0.68 [0.27–1.83]	0.72 [0.31–2.27]	0.66 [0.26–1.01]	0.37
	Hypotension MAP < P55[%]	0.0 [0.0–0.0]	0.3 [0.0-1.7]	0.0 [0.0-0.1]	0.02
	Hypotension MAP < 65[%]	7.6 [1.3–12.2]	10.2 [4.0-17.6]	2.6 [1.2–10.1]	0.07
	Hypertension systolic pressure > 140[%]	3.4 [0.7–10.2]	2.2 [0.7–7.4]	4.8 [0.6–21.3]	0.41
	Bradycardia heart rate < 45[%]	0.0 [0.0–0.0]	0.0 [0.0-0.6]	0.0 [0.0–0.0]	0.07
	Tachycardia heart rate > 90[%]	0.0 [0.0-0.2]	0.0 [0.0-0.2]	0.0 [0.0-0.2]	0.84
	Time To Extubation [HH: MM]	00:11 [00:09 − 00:18]	00:10 [00:07 − 00:15]	00:14 [00:10 − 00:19]	0.13
	Hemodynamic instability score	29 [24–36]	33 [27–43]	28 [22–33]	0.07

## Discussion

In this single-blinded, randomised, controlled trial of older patients undergoing gastrointestinal surgery, SPI-guided remifentanil administration did not affect remifentanil consumption or unwanted anaesthesia related events when compared to standard monitoring alone. Thus, the haemodynamic instability score and the incidence of low blood pressure was low in both groups. Our results are in line with those of earlies studies [20].

Invasive blood pressure monitoring is commonly used for fragile patients with co-morbidities. Interestingly, in patients who had invasive blood pressure measurement based on clinical decision, the SPI guidance resulted in higher remifentanil consumption and shorter low blood pressure times. Since the decision to insert arterial cannula was based on clinical judgement by attending anesthesiologist, these patients represent the more challenging subgroup in our study. In this subgroup, the haemodynamic instability score was markedly higher in both groups, being slightly lower numerically in the SPI group. One explanation for this might be that the opioid was given in a timely manner, thus reducing the instability of the anaesthesia. Our findings are also supported by a recent study with patient undergoing colorectal surgery under sevoflurane-remifentanil anesthesia [7]. The consumption of remifentanil was higher, but PACU treatment was not compromised. Won et al. also reported Interleukin-6 and NK cell levels which did not differ between the groups. In contrast, Funcke and colleagues [8] reported a study, where SPI had no opioid sparing effect, but the measured serum cortisol was lower when SPI was used to guide opioid administration. Based on these two controversial studies it is unclear, whether blood samples would help to grade determine the level of nociceptive reaction. One could argue that the hypotensive events were detected timelier in the invasive monitoring group. However, we verified this by only taking invasive blood pressure values every five minutes but still found a marked difference between the patient groups. Thus, we can conclude that frail patients will benefit from SPI monitoring because it allows for the timely adjustment of analgesics. Lower remifentanil consumption may not, however, be beneficial because very low remifentanil doses are associated with an increase in serum cortisol concentrations which, in turn, has multiple unwanted effects.

Previous studies have reported controversial results on the reduction of movements or unwanted responses during anesthesia [21, 22]. In a multicentre study, the number of dose adjustments as well as the amount of remifentanil was higher in the SPI group [20]. However, in those studies the patients were younger than the patients in our study. The measurement of blood pressure was also performed using intermittent measurement, indicating rather healthy patients. Therefore, we decided to include older patients to increase the likelihood of unstable anaesthesia. As described by the haemodynamics and haemodynamic instability index used in our study, anaesthesia was still quite stable in both groups. However, instability increased in the invasive blood pressure group, which is the group considered to be most at risk for anaesthesia and surgery. In this group, the percentage of low blood pressure was markedly higher in the control group, favouring the use of SPI.

In many of the published articles studying SPI, continuous infusions of remifentanil and propofol are used. However, continuous infusions often lead to otherwise stable anaesthesia, where it is harder to show the effects of the parameters when the patients are healthy. Interestingly, in studies where bolus administration of opioids is used, the result has mostly been a reduction in the amount of opioid without any negative effects on the stability of anaesthesia [23–26]. 

### Limitations

In our study, the patients were rather healthy. With healthy patients, the conduct of anaesthesia is so stable that it is hard to find any differences between study groups. However, with patients who underwent invasive blood pressure monitoring, there were differences between the treatment group and the control group. This subgroup was, however, quite small.

## Conclusion

The use of SPI did not affect the consumption of remifentanil between the control group and the SPI-guided group. Furthermore, the SPI-guided opioid administration resulted in a similar consumption of remifentanil on a group level, regardless of the type of blood pressure measurement. However, using SPI-guided remifentanil administration reduced hypotensive time in invasively monitored patients. Thus, the administration of opioids in a timely manner may benefit patients. This finding warrants further research on the invasive blood pressure monitoring of frail patients.

## Data Availability

No datasets were generated or analysed during the current study.

## Declarations

### Employment

M.S. is an employed worker of GE Healthcare Finland.
